# Self-supervised zero-shot dehazing network based on dark channel prior

**DOI:** 10.1007/s12200-023-00062-7

**Published:** 2023-04-14

**Authors:** Xinjie Xiao, Yuanhong Ren, Zhiwei Li, Nannan Zhang, Wuneng Zhou

**Affiliations:** 1 School of Electronic and Electrical Engineering, Shanghai University of Engineering Science, Shanghai, 201620 China; 2 College of Information Science and Technology, Donghua University, Shanghai, 201620 China

**Keywords:** Image dehazing, Quad-tree algorithm, Self-supervised, Zero-shot

## Abstract

Most learning-based methods previously used in image dehazing employ a supervised learning strategy, which is time-consuming and requires a large-scale dataset. However, large-scale datasets are difficult to obtain. Here, we propose a self-supervised zero-shot dehazing network (SZDNet) based on dark channel prior, which uses a hazy image generated from the output dehazed image as a pseudo-label to supervise the optimization process of the network. Additionally, we use a novel multichannel quad-tree algorithm to estimate atmospheric light values, which is more accurate than previous methods. Furthermore, the sum of the cosine distance and the mean squared error between the pseudo-label and the input image is applied as a loss function to enhance the quality of the dehazed image. The most significant advantage of the SZDNet is that it does not require a large dataset for training before performing the dehazing task. Extensive testing shows promising performances of the proposed method in both qualitative and quantitative evaluations when compared with state-of-the-art methods.

**Graphical Abstract:**

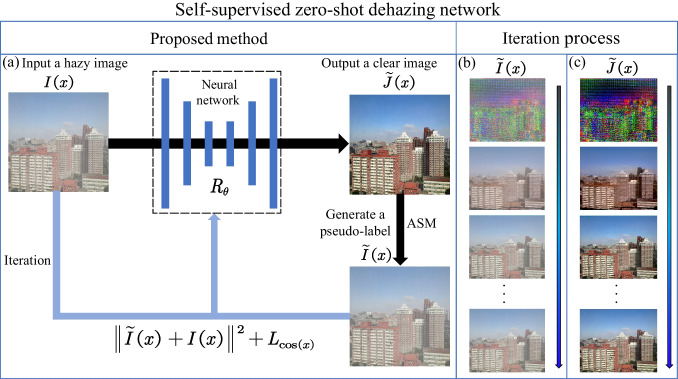

## Introduction

Haze is a special weather condition that the sky becomes blurred due to micron-sized particles suspended in the atmosphere. These suspended particles scatter and absorb light, thus deteriorating the visual clarity of an image, with image contrast degradation and color distortion. The images captured in hazy scenes significantly affect the performances of computer vision, such as target detection [1, 2] and scene understanding [3, 4]. Therefore, image dehazing has long-term importance in computer vision.

Most deep learning-based methods [5–8] for dehazing use a supervised learning strategy, which requires a large-scale dataset and corresponding ground-truth images. These methods update network parameters by calculating the loss between the image output of the dehazing network and ground-truth images. Meanwhile, network training is time-consuming and data collection is challenging. Lightweight networks [9, 10] have been proposed to reduce training time and address the issues with conventional supervised learning, by reducing the number of parameters of the neural networks (NNs). For example, Suresh et al. [10] proposed a lightweight dehazing network that uses a pre-trained teacher network to extract multi-scale information for the student dehazing network, resulting in significant parameter reduction compared to previous networks that had millions of parameters, and thus speed-up training. However, the training process of the networks remains very time-consuming and requires a large number of ground-truth images.

Self-supervised learning [11–13] has been proposed to overcome the problem with collecting ground-truth images. For instance, Wang et al. [14] proposed a physics-enhanced deep neural network that combines NNs and physical models. They fed a diffraction pattern into the NN and applied the Huygens-Fresnel principle to construct a pseudo-diffraction pattern as a label to supervise the learning process. Li et al. [15] proposed a zero-shot image dehazing (ZID) network that uses three sub-networks to generate a hazy image for guiding network training, but the dehazed images outputted by the ZID network are have low brightness and color distorted.

To address the problems in these previous methods, we propose a self-supervised zero-shot dehazing network (SZDNet) using dark channel prior. The image output of the NN is used to generate a hazy pseudo-label using the physical model. We update the NN parameters with a loss function to improve the dehazing ability. This method saves time and labor by removing the need for a large-scale dataset and ground-truth images to train the NN before using it to conduct dehazing. The physical model used by the SZDNet is the atmospheric scattering model (ASM) [16, 17], and we estimate the transmission map based on the dark channel prior (DCP) theory. He et al. [18] considered the mean of the globally brightest 0.1% pixels as the atmospheric light, but the method has a significant deviation. Therefore, Li and Zheng [19] estimated atmospheric light using a quad-tree method and achieved excellent performance. Nevertheless, this method is prone to failure in a scene with numerous white objects. To improve the precision and stability of locating the brightest sky region, we design a quad-tree algorithm with multiple channels, supported by the understanding that the sky region is primarily distributed in the top half of the image. Typical loss functions for deep learning dehazing algorithms [5, 6] include the mean-squared error (MSE) which achieves improved performance by accurately evaluating the pixel-level difference between the dehazed image output from the network and ground-truth image. However, the MSE ignores the variations between the row vectors and column vectors of two images. Thus, we improved the quality of dehazed images by adding the cosine distance to the loss function.

Our method differs considerably from the existing supervised deep learning dehazing methods [5–8], in that it has robust performances of the NNs and exhibits theoretical support from physical models. Notably, the concept of “zero-shot” is that feeding a hazy image into the NN results in a dehazed image. In brief, our method neither requires a large-scale dataset to train the NN before dehazing, nor requires the collection of corresponding haze-free images.

The main contributions of this work can be summarized as follows:

- We propose a novel self-supervised zero-shot dehazing network (SZDNet) based on DCP that outperforms some state-of-the-art (SOTA) methods.
- In accordance with the understanding that the sky region is primarily distributed in the top half of the image, we design a novel multichannel quad-tree algorithm to estimate atmospheric light values more accurately than the previous methods.
- The sum of the cosine distance and MSE is designed as the loss function of the NN, based on which the difference between pixels and the similarity between row vectors and column vectors of two images can be estimated, thereby improving the quality of the dehazed images.

## Related works

Recently, many methods for single-image dehazing have been developed, and excellent results have been obtained. These methods can be classified into prior-based [20–22] and learning-based methods [5–8].

### Prior-based image dehazing methods

Most prior-based dehazing methods use prior knowledge observed in the real world and apply them to the ASM [16, 17] for image dehazing. These methods include DCP [18], color attenuation prior [20], contrast maximization [21], and non-local prior [22]. Despite excellent performances achieved by prior-based methods, priors have some limitations because they are obtained under some assumptions and certain target scenes. For example, color attenuation prior [20] treats the scattering coefficient that varies with the scene depth as a constant, thereby affecting dehazing in some cases.

### Learning-based image dehazing methods

Learning-based dehazing methods have been developed recently and achieved excellent dehazing results. Learning-based methods can be classified into two groups: The first group integrates the ASM and estimate the dehazing process parameters using NN [5, 6]. The second group learns straight mapping from hazy-to-clear images [7, 8]. For example, DehazeNet [5] combined with an ASM took hazy images as the input and estimated the medium transmission map using a NN to restore dehazed images. The enhanced pix2pix dehazing network (EPDN) [8] followed the hazy-to-clear image translation approach and generated dehazed images without relying on ASMs. However, it is difficult and expensive to collect large-scale datasets and ground-truth images sufficient for such learning-based dehazing methods.

### Advances in self-supervised learning methods

Self-supervised learning methods [11–15] have made remarkable achievements in image processing because they do not require manual labels. For example, Hendriksen et al. [12] proposed a novel “Noise2Inverse” strategy which uses the noise model to compute multiple statistically independent reconstructions. This method uses self-supervised denoising to obtain improved denoising performance. In Ref. [13], Chen et al. proposed a context restoration strategy to better utilize unlabelled images. Self-supervised learning methods are independent of ground-truth images and have achieved promising performances. Although the self-supervised learning method is widely used in many other fields, it is rarely used in image dehazing. A ZID network [15] comprising three joint subnetworks to disentangle the input hazy image was recently proposed and has achieved excellent dehazing performance. However, the dehazed image using ZID has low brightness.

The SZDNet proposed in this study differs from these methods and does not require a large dataset for training. We only used the U-Net [23] as the backbone of the network. First, we fed a hazy image into the network, and the hazy pseudo-label was generated by processing the output of the U-Net. Then, we optimized the parameters of U-Net using the loss function. Experimental results showed that SZDNet achieved better performance than some SOTA methods.

## Proposed method

The network architecture of SZDNet and its various components are described first in this section. Then, we introduce a multichannel quad-tree algorithm to find the atmospheric light value* A*, and finally, we construct the loss function to update the weights and biases.

### SZDNet system architecture

**Fig. 1:**
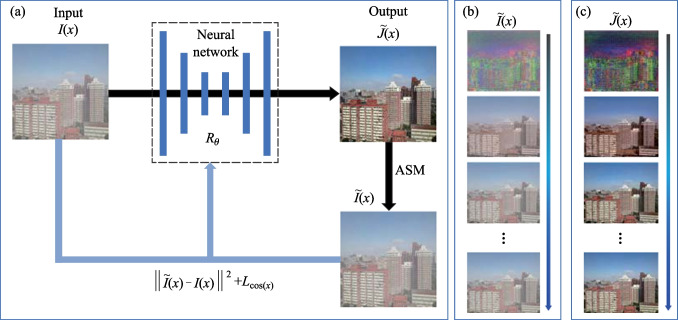
Principle and architecture diagram of the SZDNet. **a** A hazy image $I\left( x \right)$ is fed into the neural network, and a dehazed image $\tilde{J}\left( x \right)$ is output after processing by the neural network, which is numerically treated to simulate the hazy image generation processes through the ASM to generate $\tilde{I}\left( x \right)$.We use the sum of the MSE and the cosine distance ($L_{\cos (x)}$) between $I\left( x \right)$ and $\tilde{I}\left( x \right)$ as the loss function to update the parameter $R_{\theta }$ of the neural network. **b** Evolution of $\tilde{I}\left( x \right)$ during the optimization process. **c** Evolution of $\tilde{J}\left( x \right)$ during the optimization process

Many image dehazing methods [5–8] make use of supervised learning with large-scale datasets. However, acquiring large datasets and ground-truth images is challenging. To address this problem, we proposed a self-supervised zero-shot dehazing network (SZDNet) using DCP. The system architecture and basic principle are schematically outlined in Fig. 1a. Here, we use the U-Net as the NN. First, we fed a hazy image $I\left( x \right)$ into the NN to generate $\tilde{J}\left( x \right)$ after NN processing. In conventional supervised dehazing NNs, the NN parameters are optimized using the loss between $\tilde{J}\left( x \right)$ and ground-truth image $J\left( x \right)$. The proposed SZDNet does not rely on ground-truth images, but it uses the DCP and the multichannel quad-tree algorithm to derive the transmission map $t\left( x \right)$ and the atmospheric light value *A*, respectively. The principle will be expressed in the next section. We fed $\tilde{J}\left( x \right)$ into a physical ASM, generating a hazy pseudo-label $\tilde{I}\left( x \right)$. The loss between the pseudo-label $\tilde{I}\left( x \right)$ and the input image $I\left( x \right)$ optimizes the network parameters, forcing the generated pseudo-label $\tilde{I}\left( x \right)$ to converge to $I\left( x \right)$. Figure 1b shows the optimization process for the pseudo-label $\tilde{I}\left( x \right)$. With the iterative process, the optimized dehazed image $\tilde{J}\left( x \right)$ converges to a near-optimal solution. Figure 1c shows the optimization process of the dehazed image. In summary, the proposed method adopts zero-shot learning and removes haze using only one hazy image.

**Fig. 2:**
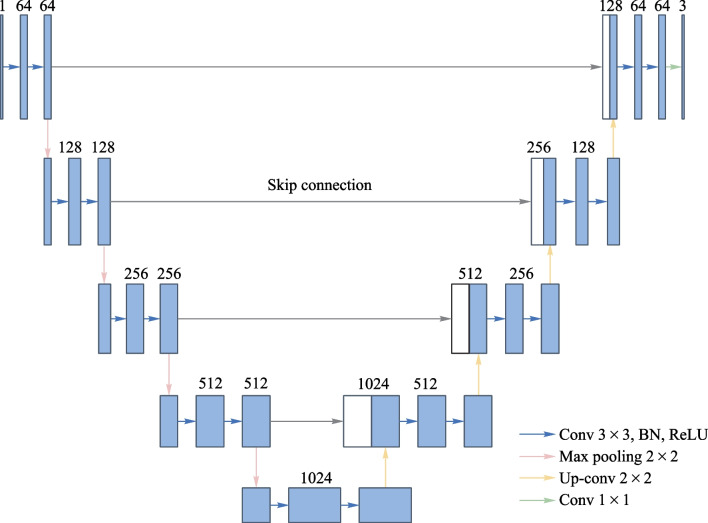
Architecture diagram of the U-Net

U-Net has encoders and decoders and can capture a lot of spatial information. Here, the main function of U-Net is to extract image features, reconstruct clear images and refine the clear images using loss function through iterative optimization. The architecture of U-Net network is shown in Fig. 2, which consists of encoders and decoders. In the encoder network, with each down-sampling operation, the image size is reduced by half and the dimensionality is doubled. By repeating this operation, the higher-level features of the image can be fully extracted and the redundant information can be filtered out. In the decoder network, for each up-sampling operation, the image size is doubled and dimensionality is halved. A skip connection structure is used between the corresponding stages of the encoder and decoder to supplement the low-level feature information and to better restore the image detail information.

Below, we introduce the technical details of the SZDNet. In computer vision and graphics, the ASM [16, 17] accounts for hazy image generation. Its mathematical model is given as

1 $$I\left( x \right) = J\left( x \right)t\left( x \right) + A\left( {1 - t\left( x \right)} \right) = ASM\left( {J\left( x \right)} \right),$$

where $x$ denotes the spatial coordinate, $I\left( x \right)$ is the hazy image, $J\left( x \right)$ is the haze-free ground-truth image, $t\left( x \right)$ is the transmission map, $A$ is the atmospheric light, and $ASM\left( \cdot \right)$ denotes the mapping function from the haze-free ground-truth image $J\left( x \right)$ to the corresponding hazy image $I\left( x \right)$.

From the ASM, we obtain $J\left( x \right)$ as

2 $$J\left( x \right) = ASM^{ - 1} \left( {I\left( x \right)} \right){ = }\frac{{I\left( x \right) - A\left( {1 - t\left( x \right)} \right)}}{t\left( x \right)}.$$

The transmission map obtained by the DCP is given by

3 $$t_{1} = 1 - w\mathop {\min }\limits_{y \in \Omega \left( x \right)} \left[ {\mathop {\min }\limits_{c} \frac{{I^{c} \left( x \right)}}{{A^{c} }}} \right],$$

4 $$t\left( x \right) = G\left( {t_{1} \left( x \right)} \right),$$

where $t_{1}$ is the coarse transmission map, $w$ is the correction factor and $0 < w \le 1$, $\Omega \left( x \right)$ denotes the set of spatial coordinates $x$, $c$ denotes the three color channels R, G, and B, and $G\left( \cdot \right)$ denotes the guide filter operator [24, 25] that can refine the transmission map.

In the proposed method, the atmospheric light $A$ is obtained using a multichannel quad-tree search algorithm described in the next section, and the mathematical formula for the pseudo-label $\tilde{I}\left( x \right)$ is given by

5 $$\tilde{I}\left( x \right) = \tilde{J}\left( x \right)t\left( x \right) + A\left( {1 - t\left( x \right)} \right) = ASM\left( {\tilde{J}\left( x \right)} \right),$$

where $\tilde{J}\left( x \right)$ denotes the dehazed image output from the neural network.

### Estimation of atmospheric light value

**Fig. 3:**
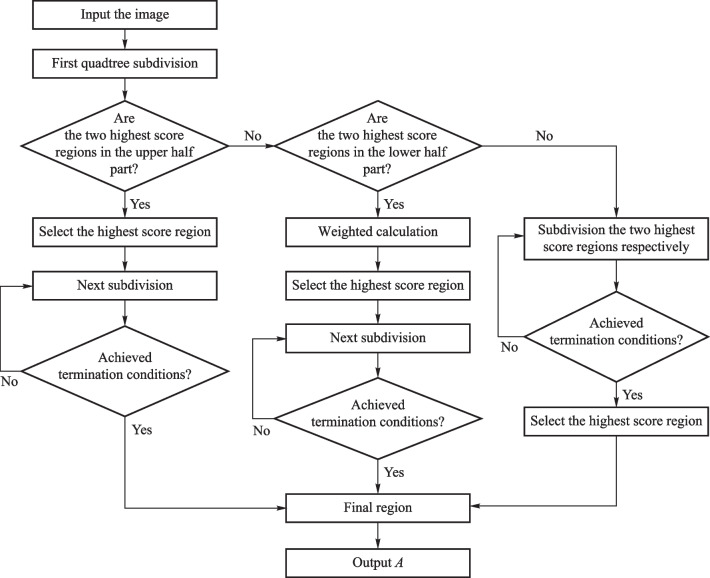
Flowchart of multichannel quad-tree algorithm to estimate atmospheric light value

Previous dehazing algorithms focused on optimizing the transmission map, while ignoring the importance of the atmospheric light value. Since a large amount of haze increases the brightness of the scene target, Tan [21] used the maximum pixel value of the dense haze region in the image as the atmospheric light value, considering the characteristics of haze. He et al. [18] applied the mean of the globally brightest 0.1% pixels as the atmospheric light value. Li and Zheng [19] proposed a quad-tree method to estimate the atmospheric light value. Although their methods improved the accuracy of estimating atmospheric light values, some deviations exist for scenes with several white objects, and sometimes white objects are selected instead of the brightest areas of the sky. Inspired by the work of Wang et al. [26], and considering the results from the quad-tree search algorithm and the prior knowledge that the sky region lies on the upper part of an image, we developed a multichannel quad-tree search algorithm to improve the precision and stability of locating the brightest sky region. Figure 3 shows the flowchart of the multichannel quad-tree algorithm. The image is divided equally into four regions $t_{n}^{i}$, $i \in \left\{ {1,2,3,4} \right\}$ representing the left and right quarters of the top and bottom halves, respectively. $n$ denotes the number of division level, $n = 1$ is the first division. We define $I\left( {t_{n}^{i} } \right)$ as a matrix that contains the corresponding gray values for each pixel in region $t_{n}^{i}$, and use the average of $I\left( {t_{n}^{i} } \right)$ as the score $M\left( {t_{n}^{i} } \right)$ for this region, as shown in the equation below:

6 $$M\left( {t_{n}^{i} } \right) = {\text{mean}}\left( {I\left( {t_{n}^{i} } \right)} \right).$$

Suppose the two regions with the highest scores after the initial division are in the upper half of the image region $\left( {t_{1}^{1} ,\;t_{1}^{2} } \right)$, then the region with the highest score is considered the part to be processed in the next iteration. This is done by dividing itinto smaller blocks and the quad-tree decomposition method and Eq. (6) are used to calculate the scores. The iteration continues and terminates when the score becomes smaller than the predefined threshold, to get the final region $t_{{{\text{final}}}}$. If the two regions with the highest scores after the initial division are in the bottom half region $\left( {t_{1}^{3} ,\;t_{1}^{4} } \right)$ of the image, the upper half region must be weighted. Suppose the weighting factor is $e\left( {e > 1} \right)$, then, the scores of $\left\{ {e \times M\left( {t_{1}^{1} } \right),\;e \times M\left( {t_{1}^{2} } \right),\;M\left( {t_{1}^{3} } \right),\;M\left( {t_{1}^{4} } \right)} \right\}$ are compared, and the selected region with highest score continues to be subdivided accordingly using the quad-tree method until the termination condition is reached to obtain $t_{{{\text{final}}}}$. Otherwise, the two highest-scoring regions are subdivided respectively, and the highest final score region is chosen as $t_{{{\text{final}}}}$ when the termination condition is met.

The quad-tree subdivision process has a termination condition that states that the iteration terminates if the difference between the highest and the second-highest scores is less than $M_{T}$ or the width of the highest score region is less than $w$. The termination conditions are expressed as

7 $$\min \left| {M\left( {t_{n}^{k} } \right) - M\left( {t_{n}^{m} } \right)} \right| < M_{T} ,$$

or

8 $$W\left( {t_{n}^{k} } \right) < w,$$

where $\min$ is the minimum operator, $M\left( {t_{n}^{k} } \right)$ denotes the highest score, $M\left( {t_{n}^{m} } \right)$ denotes the second-highest score, and $W\left( {t_{n}^{k} } \right)$ is the width of the region with the highest score. From the experimental data, we set $e = 1.1,\;M_{T} = 1,\;w = 25$ for image dehazing.

### Loss function for SZDNet

**Fig. 4:**
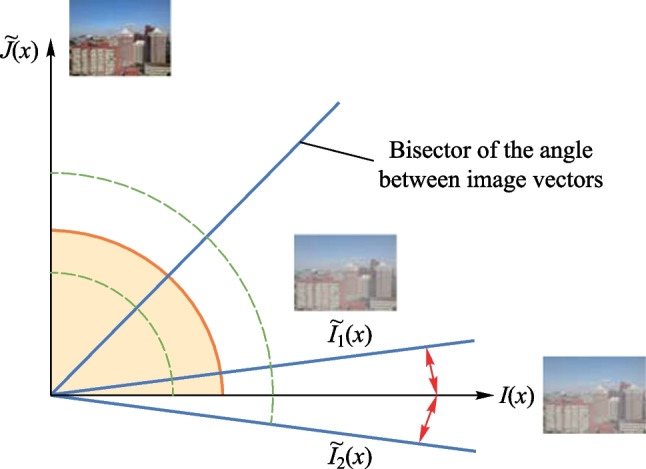
Cosine similarity between the hazy image and the pseudo-label

MSE loss function is an effective tool employed by the conventional supervised learning dehazing network. However, it only evaluates pixel-level changes and does not examine variations between the row vectors and column vectors of hazy and ground-truth images. Gao et al. [27] used the cosine similarity between the generated and target images to represent the similarity between the identity information of both images, thus to solve the problem of determining how to swap the identity information when dealing with the face-swapping task. Inspired by their work, we introduced the cosine distance into the loss function in this study to address the limitations of MSE loss function. Figure 4 shows the cosine similarity between the hazy image and the pseudo-label. The horizontal and vertical axes in Fig. 4 represent the input hazy image $I\left( x \right)$ and the dehazed image $\tilde{J}\left( x \right)$ at the output of NN, respectively. The yellow region marked the case that the angle between $I\left( x \right)$ and $\tilde{J}\left( x \right)$ is a right angle and the cosine similarity is 0. The overlap of the two sector regions marked by the green dashed curves indicate that the generated pseudo-label $\tilde{I}\left( x \right)$ is near the angle bisector between $I\left( x \right)$ and $\tilde{J}\left( x \right)$, and is not similar to $I\left( x \right)$.

A high quality pseudo-label $\tilde{I}\left( x \right)$ should be close enough to the input hazy image $I\left( x \right)$, thus should be within a small region centered on $I\left( x \right)$, eg., between $\tilde{I}_{{1}} \left( x \right)$ and $\tilde{I}_{{2}} \left( x \right)$ as shown in Fig. 4. Here, the cosines of $\tilde{I}_{{1}} \left( x \right)$ and $\tilde{I}_{{2}} \left( x \right)$ with $I\left( x \right)$ are 0.9.

Thus, the loss function is as follows

9 $$Loss = \;\;\left\| {\tilde{I}\left( x \right) - I\left( x \right)} \right\|^{2} + L_{\cos \left( x \right)} ,$$

10 $$L_{\cos \left( x \right)} = 1 - \cos \left( {I\left( x \right),\;\tilde{I}\left( x \right)} \right),$$

where $L_{\cos \left( x \right)}$ and $\left\| {\tilde{I}\left( x \right) - I\left( x \right)} \right\|^{2}$ denote the cosine distance and the MSE, respectively. $\cos \left( {I\left( x \right),\;\tilde{I}\left( x \right)} \right)$ denotes the cosine similarity between $I\left( x \right)$ and $\tilde{I}\left( x \right)$. In the optimization process, the more similar $\tilde{I}\left( x \right)$ is to $I\left( x \right)$, the more the $\cos \left( {I\left( x \right),\;\tilde{I}\left( x \right)} \right)$ converges to 1, the smaller the $L_{\cos \left( x \right)}$, and the smaller the loss function.

## Experiments

We experimented the proposed method using three datasets and compared the results with the SOTA methods based on four evaluation metrics. The experimental settings are given first in this section. Next, we demonstrate the superiority of the multichannel quad-tree search algorithm in estimating atmospheric light *A*. We show quantitative and qualitative results on the three datasets, and finally conduct an ablation experiment.

### Experiment settings

This section presents the datasets used, the evaluation metrics, and the implementation details.

#### Datasets

Our method was evaluated on two real-world datasets and one synthetic dataset. For the synthetic dataset, we employed the synthetic objective testing set (SOTS) from a large-scale benchmark REalistic Single Image DEhazing (RESIDE) [28], which contains 1000 hazy images and corresponding ground-truth images. We randomly selected 100 pairs of images from SOTS for testing. For the real-world datasets, we employed 35 and 45 hazy images and the corresponding ground-truth images from I-HAZE [29] and O-HAZE [30] benchmark, respectively. The hazy images of the real-world datasets were generated using real and machine haze. Also, both the images with and without the haze were captured under the same lighting conditions.

#### Evaluation metrics

To evaluate the performance of the proposed SZDNet, we employed natural image quality evaluator (NIQE) [31], fog aware density evaluator (FADE), peak signal-to-noise ratio (PSNR), and structural similarity (SSIM), all of which are common evaluation metrics in image dehazing. FADE and NIQE are non-reference metrics, whereas PSNR and SSIM were compared with ground-truth images. A smaller FADE and NIQE represent less haze residue and higher perceptual quality, respectively. We compared the proposed method with other prior-based methods (e.g., DCP [18], dark direct attenuation prior (DDAP) [32]), methods based on enhanced atmospheric scattering model (e.g., image dehazing and exposure (IDE) using an enhanced atmospheric scattering model [33]), supervised learning-based methods (e.g., DehazeNet [5], AOD-Net [6], EPDN [8], TCN [34]), and unsupervised learning-based methods (e.g., RefineDNet [35], and USID-Net [36]).

#### Implementation details

The experiments were conducted on a computer with Inter Core i7-11700K and NVIDIA GeForce RTX 3090. The NN was implemented with TensorFlow version 1.15 using Python 3.6.0 on an Ubuntu 20.04.1 LTS system. The training process gets the dehazed result directly from single frame training and in-depth studies reveal that the quality of the dehazed images reached a desirable performance when the number of iterations reaches 1000. The total time consumption that the proposed method to process 1000 hazy images is 44,000 s, and the average time consumption of processing each image is 44 s. We optimized the weights and biases of the NN using the Adam optimizer [37] and an initial learning rate of 0.001. Also, we added a uniformly distributed noise of 0 to 1/30 for better conversion [38]. Furthermore, we used an exponential-decay strategy with a decay rate of 0.95 to adjust the learning rate. We removed the noise and generated a dehazed image to complete the optimization.

### Comparison of atmospheric light estimation

**Fig. 5:**
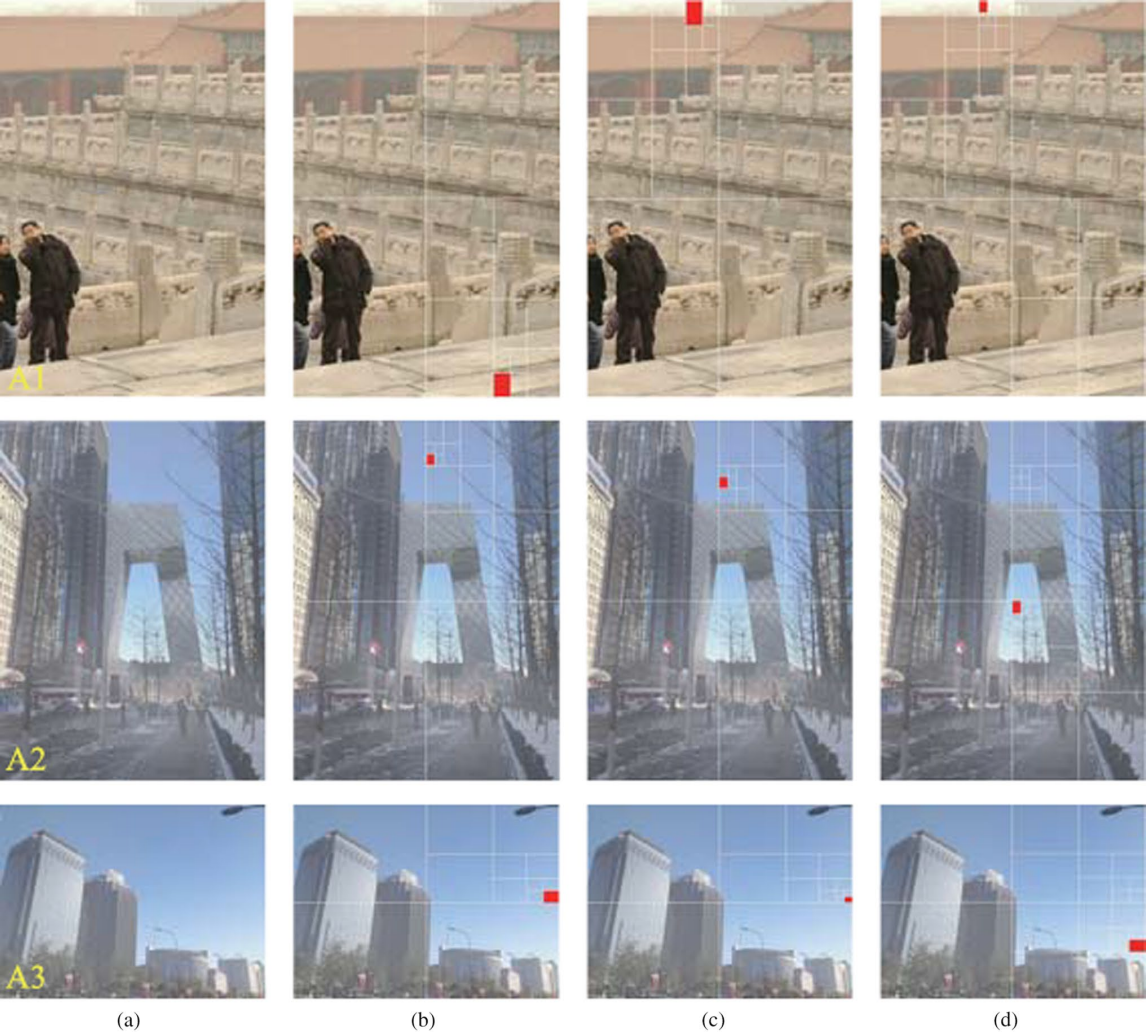
Comparison of atmospheric light estimation with SOTA methods. **a** Hazy images. **b**–**d** Brightest sky regions (marked by red rectangles) obtained using the method reported by (**b**) Li et al. [19], (**c**) Wang et al. [26], and (**d**) proposed method

The dehazing results are positively impacted by the application of an accurate atmospheric light value. We developed a multichannel quad-tree search algorithm that improves the accuracy and robustness of locating the brightest sky region and estimates the atmospheric light values. We compared our results with that of the algorithms reported in Refs. [19] and [26], as shown in Fig. 5, where three experimental images A1, A2 and A3 are used and the marked red regions represent the brightest sky regions searched out. The method in Ref. [19] achieves excellent performance but fails to select the correct sky region when the image contains several white objects (see image A1 in Fig. 5b). The algorithm in [26] can locate the sky region, but not the brightest sky region (see images A2 and A3 in Fig. 5c). Our method outperformed these methods by accurately locating the brightest sky region (Fig. 5d). Moreover, our multichannel quad-tree search algorithm considers an image with the sky as the main component, therefore, it still works even the brightest sky region is located in the lower middle of the image.

### Comparisons on a synthetic dataset

**Fig. 6:**
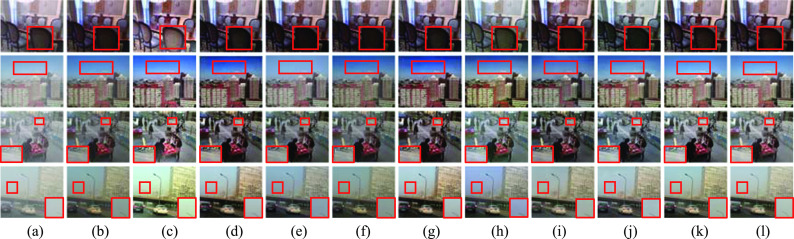
SZDNet and well-known methods were compared qualitatively on the SOTS dataset. **a** Hazy images. **b** DCP [18]. **c** IDE [33]. **d** DDAP [32]. **e** DehazeNet [5]. **f** AOD-Net [6]. **g** EPDN [8]. **h** TCN [34]. **i** RefineDNet [35]. **j** USID-Net [36]. **k** SZDNet. **l** Ground-truth. Red rectangles indicate certain areas that are suggested to be viewed in closer detail for improved visualization and comparison

Table 1 shows the quantitative comparison on the SOTS dataset, and Fig. 6 shows the corresponding qualitative comparison. Table 1 summarizes the metrics compared with some SOTA methods using a test set of 100 randomly selected images from SOTS of RESIDE. The results show that SZDNet is superior to all methods in PSNR and SSIM, and achieves the second-best performance in FADE compared with SOTA methods. Although SZDNet is slightly inferior to IDE, DDAP, TCN, and RefineDNet in NIQE, it demonstrates the best visual performance in Fig. 6. For example, compared to the ground-truth images, IDE and TCN showed color distortions despite achieving satisfactory visual results (Fig. 6c, h). The dehazed images output by DDAP and RefineDNet are too low brightness (Fig. 6d, i). Figure 6k shows the recovered results using SZDNet. The images recovered by SZDNet have better visibility and are similar to the ground-truth images. We conclude that more attention should be paid to the results of comparison with ground-truth images for a dehazing task. By combining the results of the four evaluation metrics, SZDNet achieved a more satisfactory performance.

**Table 1:**
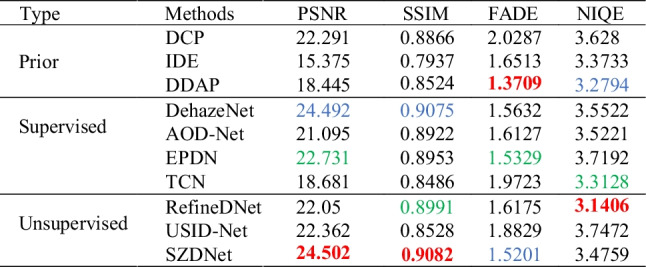
Quantitative comparison between SZDNet and SOTA methods on the SOTS dataset. The best, second best, and third best performances are marked in red, blue, and green, respectively

### Comparisons on real-world datasets

Tables 2 shows the quantitative comparison of SZDNet and the SOTA methods, on the I-HAZE and O-HAZE datasets. From Table 2, SZDNet achieved the best performance in PSNR and SSIM on the test dataset I-HAZE compared with the SOTA methods. The SZDNet achieved the best and second-best results in SSIM and NIQE compared with SOTA methods on the test dataset O-HAZE. SZDNet achieved better performance because atmospheric light values can be estimated more accurately, and including the cosine distance in the loss function reduces the difference between the hazy input images and pseudo-label images.

**Table 2:**
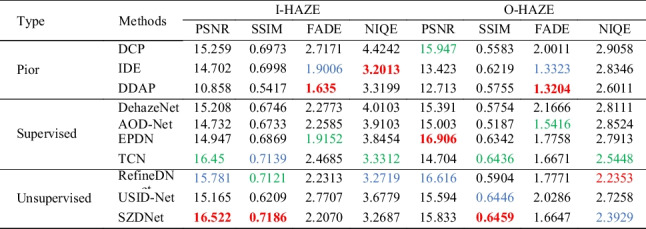
Quantitative comparison between SZDnet and SOTA methods on the I-haze and O-haze datasets. The best, second best, and third best performances are marked in red, blue, and green, respectively

**Fig. 7:**
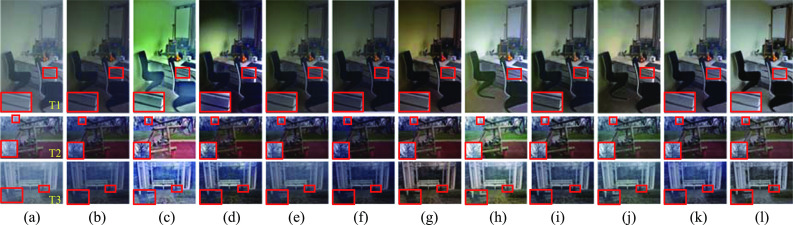
SZDNet and well-known methods were compared qualitatively on real-world datasets. **a** Hazy images. **b** DCP [18]. **c** IDE [33]. **d** DDAP [32]. **e** DehazeNet [5]. **f** AOD-Net [6]. **g** EPDN [8]. **h** TCN [34]. **i** RefineDNet [35]. **j** USID-Net [36]. **k** SZDNet. **l** Ground-truth. Red rectangles indicate certain areas that are suggested to be viewed in closer detail for improved visualization and comparison

Additionally, the quantitative and qualitative comparison results are further displayed for three selected experimental images T1, T2, and T3. Figure 7 shows that the dehazed images with DCP [18], DDAP [32], DehazeNet [5], and AOD-Net [6] had lower brightness compared with the ground-truth images (see T1, Fig. 7b, d–f). The dehazed images with IDE [33], EPDN [8], TCN [34], RefineDNet [35], and USID-Net [36] showed color distortion (Fig. 7c, g–j). SZDNet performed better in dehazing, and the restored images were the most similar to the ground-truth images (T1 in Fig. 7k, l). Also, we specifed the corresponding performance metrics in Table 3, showing that the proposed SZDNet achieved the best or the second-best results in PSNR and SSIM except for T2. FADE scores of our method are close to the best methods. Despite IDE, DDAP, and AOD-Net obtaining better FADE scores, the dehazed images were color distorted or have low brightness (T1 and T3 in Fig. 7c, d, f). Thus, FADE is not the most appropriate evaluation metric. Although NIQE of our method was slightly inferior compared to other methods, our dehazed results were the closest to the ground-truth images. Upon combining all four evaluation metrics and the qualitative comparisons, SZDNet achieved a more satisfactory performance.

**Table 3:**
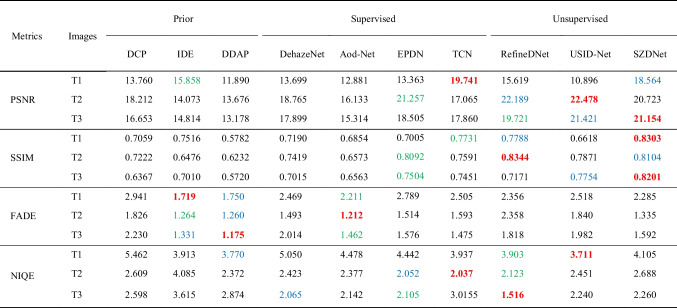
Qualitative comparison between SZDNet and the SOTA dehazing method on images. The best, second best, and third best performances are marked in red, blue, and green, respectively

### Comparisons on the real-shot images

**Fig. 8:**
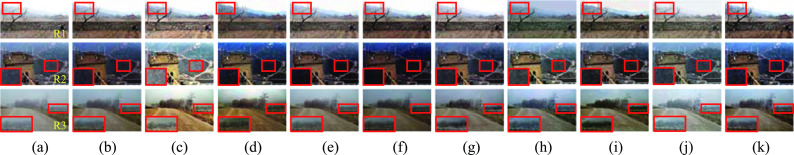
SZDNet and well-known methods were compared qualitatively on the real shot images. **a** Hazy images. **b** DCP [18]. **c** IDE [33]. **d** DDAP [32]. **e** DehazeNet [5]. **f** AOD-Net [6]. **g** EPDN [8]. **h** TCN [34]. (i) RefineDNet [35]. **j** USID-Net [36]. **k** SZDNet. Red rectangles indicate certain areas that are suggested to be viewed in closer detail for improved visualization and comparison

As NN trained using the supervised approach perform better on the synthetic dataset than on real-shot images, we further compared SZDNet with the prior-based and learning-based approaches on the real-shot images. We did not quantitatively compare the dehazed images in Fig. 8 because the ground-truth images of real-shot hazy images were unavailable.

Figure 8 shows that all methods perform well on image R1. The dehazing effect in the marked region in the upper left corner with DCP, IDE, DehazeNet, AOD-Net, EPDN, TCN, and USID-Net was not good, whereas images processed by DDAP, RefineDNet, and the proposed SZDNet had better visibility and dehazing effect (see image R1 in Fig. 8d, i, k). As shown by the red rectangular region in image R2 of Fig. 8b, d–g, contrast of the trees in the middle of the image is sacrificed with DCP, DDAP, DehazeNet, AOD-Net, and EPDN.For IDE, the entire image showed color distortion. TCN, RefineDNet, and USID-Net could not entirely remove the haze (Fig. 8c, h–j). However, as observed in Fig. 8k, SZDNet performed the best compared with the other methods and the trees in the middle region are visible. In the marked red regions in image R3 (Fig. 8), the trees were the clearest with the SZDNet (Fig. 8k). In contrast, the brightness with DCP and AOD-Net were low that with IDE was too large, the dehazed image with DehazeNet and USID-Net had residual haze on the image, and the trees in the middle of the dehazed image were distorted with DDAP, EPDN and RefineDNet (see image R3). Thus, the dehazed images with SZDNet were the closest to the original image and had better dehazing results even with a self-supervised and zero-shot method.

### Ablation study

We performed ablation tests by analyzing various factors, including a multichannel quad-tree algorithm to estimate the atmospheric light and cosine distance ($L_{\cos \left( x \right)}$) to show the efficiency of the proposed SZDNet.

We built a basic network as the baseline for reference, where the atmospheric light and transmission maps are estimated with DCP, dehazed images are obtained with U-net, and MSE are chosen as the loss function. We added the following modules to the basic dehazing network: (1) A(quad-tree), where a multichannel quad-tree approach was exploited to solve atmospheric light instead of using the mean of the globally brightest $0.1\%$ pixels. (2) A(quad-tree) + $L_{\cos \left( x \right)}$, where the cosine distance ($L_{\cos \left( x \right)}$) was added to the loss function besides (1), which is the so-called SZDNet proposed in this work. Table 4 shows the specific performances for 100 randomly selected images from SOTS of RESIDE.

**Table 4:** Results on the SOTS dataset

Method	PSNR	SSIM	FADE	NIQE
Baseline	23.251	0.8946	**1.4904**	3.8670
Baseline + A(quad-tree)	23.924	0.8954	1.4914	3.8569
SZDNet	**24.502**	**0.9082**	1.5201	**3.4759**

The bold numbers denote the best value in each category

#### Estimating atmospheric light with a multichannel quad-tree algorithm

The atmospheric light value obtained by averaging the brightest $0.1\%$ pixels varied significantly. The dehazing network performed better when the atmospheric light was detected by multichannel quad-tree method because it could accurately pinpoint the brightest sky region. Table 4 shows that SZDNet outperformed the basic dehazing network on PSNR, SSIM, and NIQE. Thus, the multichannel quad-tree algorithm for solving atmospheric light significantly improved dehazing effect.

#### Cosine distance ($L_{\cos \left( x \right)}$)

**Fig. 9:**
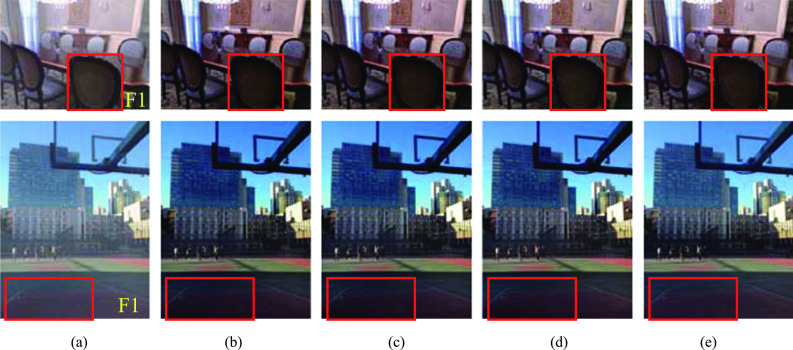
Qualitative comparison between the proposed SZDNet and the other two methods (baseline and baseline + A(quad-tree)) in the two example images from SOTS Dataset. **a** Input hazy image. **b** Baseline. **c** Baseline + A(quad-tree). **d** SZDNet. **e** Ground-truth images

Although MSE is the mostly used loss function in dehazing, it ignores the link between neighboring pixels. The row vectors and column vectors of two images were optimized when the cosine distance was added to the loss function, by examining the relationship between neighboring pixels. Table 4 shows that PSNR, SSIM, and NIQE of SZDNet were better than (2), whereas the FADE was slightly worse. The reason for the latter can be attributed to the fact that FADE might mistake the recovered shadow details for haze residue. We used two images from SOTS for further testing, and the result was given in Fig. 9 and Table 5. Despite the inferior FADE scores of the proposed SZDNet, our results outperformed the other methods and the dehazed images were closer to the ground-truth images. Figure 9d, e show that the shaded regions marked by red rectangulars in our results were similar to the ground-truth images. The shaded regions in the other results have low brightness although the FADE scores were better (image F1 and F2 in Fig. 9b, c). This results showed that adding the cosine distance to the loss function improved dehazing effect.

**Table 5:** Quantitative comparison between the proposed SZDNet and the other two methods

Images	Baseline	Baseline + A(quad-tree)	SZDNet	Ground-truth
F1	1.3929	1.3998	1.5498	1.6774
F2	1.4461	1.4415	1.4914	1.6741

## Conclusions

This study proposed a novel self-supervised zero-shot dehazing network combining the U-Net and ASM. A new multichannel quad-tree algorithm was developed to estimate the atmospheric light value, which outperformed previous methods in terms of accuracy and robustness in localizing the brightest sky region. The cosine distance was introduced to the MSE as the loss function to consider the entirety of all pixels. SZDNet had the advantage of generating a dehazed image by simply inputting a hazy image without dataset training. Using synthetic and actual datasets, we conducted experiments to demonstrate the dehazing ability of SZDNet. The results showed that the SZDNet outperformed other SOTA methods. The SZDNet might perform better if a more effective technique can be developed to infer the transmission map and the atmospheric light value, which will be addressed in our future research.

## Data Availability

The data that support the findings of this study are available from the corresponding author, upon reasonable request.
